# Neurological Emergencies

**DOI:** 10.1155/2012/208193

**Published:** 2012-10-14

**Authors:** Joseph R. Shiber, Chamisa Macindoe, Oliver Flower, William A. Knight IV, Julian Bösel

**Affiliations:** ^1^Departments of Emergency Medicine and Critical Care College of Medicine, University of Florida, Jacksonville, FL 32209, USA; ^2^Departments of Emergency Medicine and Surgery, University of New Mexico, Albuquerque, NM 87131, USA; ^3^Department of Intensive Care Medicine, E25—Royal North Shore Hospital, The University of Sydney, Sydney, NSW 2006, Australia; ^4^Departments of Emergency Medicine and Neurosurgery, University of Cincinnati, Cincinnati, OH 45267-0769, USA; ^5^Neurological Clinic, University of Heidelberg, Im Neuenheimer Feld 400, 69120 Heidelberg, Germany

Emergency departments (EDs) are the typical initial contact for seriously ill and injured patients. Although diverse and sometimes subtle in presentation, acute neurological diseases have an urgency that makes their rapid diagnosis and treatment crucial for improving outcomes. These patients may need to go to the intensive care unit, the interventional radiology suite, or the operating room and may require various consulting services but all of their care begins with the emergency physician (EP). It is therefore vital that EPs have expertise in recognizing these disorders and rapidly initiating appropriate treatments.

Investigators and clinicians from around the world submitted manuscripts for consideration in this special issue focusing on neurological emergencies. We are pleased to present this special issue in order to stimulate international dialogue and advance efforts for improving the diagnosis, close monitoring, and initial treatment of these potentially devastating disorders.

This special issue includes a significant article by Dr. L. H. Tan and Dr. O. Flower from Royal North Shore Hospital, Australia on “*Reversible cerebral vasoconstriction syndrome: An important cause of acute severe headache*” the authors describe the epidemiology, pathophysiology, clinical and diagnostic features, and a summary of the treatments of this under-recognized syndrome. Dr. C. Tacon from Sydney Children's Hospital, Australia and Dr. O. Flower present an excellent review on “*Diagnosis and management of bacterial meningitis in the paediatric population*” it discusses the changing epidemiology due to new childhood vaccines, current laboratory testing, and clinical tools that can aid in diagnosing bacterial meningitis, as well as the controversies and advances in patient management. Dr. C. McDermott and Dr. N. Collins from University College Dublin, Ireland offer a noteworthy research article “*Prehospital medication administration: A randomised study comparing intranasal and intravenous routes*” the authors investigate the safety and effectiveness of intranasal naloxone administration by paramedic trainees. An indispensable review on “*Sedation in traumatic brain injury*” by Dr. O. Flower and Dr. S. Hellings from Royal North Shore Hospital, Australia presents and compares the medication choices using evidence-based support for recommendations on the optimal clinical context for the use of each agent. Lastly, Dr. J. V. Pope and Dr. J. A. Edlow from Harvard Medical School, USA present an outstanding article “*Avoiding misdiagnosis in patients with neurologic emergencies*” this valuable paper is intended to assist EPs and other healthcare providers in understanding how diagnostic errors occur in order to make an accurate diagnosis and improve patient care for the common presenting complaints of headache, dizziness, back pain, weakness, and seizure.

*Joseph R. Shiber* *Joseph R. Shiber* *Chamisa Macindoe* *Chamisa Macindoe* *Oliver Flower* *Oliver Flower* *William A. Knight IV* *William A. Knight IV* *Julian Bösel* *Julian Bösel*

